# Use of ZnAl-Layered Double Hydroxide (LDH) to Extend the Service Life of Reinforced Concrete

**DOI:** 10.3390/ma13071769

**Published:** 2020-04-09

**Authors:** Celestino Gomes, Zahid Mir, Rui Sampaio, Alexandre Bastos, João Tedim, Frederico Maia, Cláudia Rocha, Mário Ferreira

**Affiliations:** 1 DEMaC—Department of Materials and Ceramic Engineering, Universidade de Aveiro, 3810-193 Aveiro, Portugal; jcv.gomes@ua.pt (C.G.); ruisampaio@ua.pt (R.S.); joao.tedim@ua.pt (J.T.); mgferreira@ua.pt (M.F.); 2 CICECO—Aveiro Institute of Materials, Universidade de Aveiro, 3810-193 Aveiro, Portugal; 3 Institute of Materials Research, Helmholtz-Zentrum Geesthacht, Max-Planck-Str. 1, 21502 Geesthacht, Germany; Zahid.Mir@hzg.de; 4 Smallmatek—Small Materials and Technologies, Lda, 3810-075 Aveiro, Portugal; frederico.maia@smallmatek.pt (F.M.); claudia.rocha@smallmatek.pt (C.R.)

**Keywords:** LDH, layered double hydroxide, corrosion, concrete

## Abstract

This work investigated the use of ZnAl-layered double hydroxide (LDH) intercalated with nitrate or nitrite ions for controlling the corrosion of steel in reinforced concrete. The work started by analyzing the stability of the powder in the 1–14 pH range and the capacity for capturing chloride ions in aqueous solutions of different pH. The effect of the ZnAl-LDH on the corrosion of steel was studied in aqueous 0.05 M NaCl solution and in mortars immersed in 3.5% NaCl. It was found that the LDH powders dissolved partially at pH > 12. The LDH was able to capture chloride ions from the external solution, but the process was pH-dependent and stopped at high pH due to the partial dissolution of LDH and the preferential exchange of OH^–^ ions. These results seemed to imply that ZnAl-LDH would not work in the alkaline environment inside the concrete. Nonetheless, preliminary results with mortars containing ZnAl-LDH showed lower penetration of chloride ions and higher corrosion resistance of the steel rebars.

## 1. Introduction

Reinforced concrete is the most common construction material in the world. It is a composite material, combining the high resistance to compression of hardened concrete with the high tensile strength and ductility of steel. Concrete and steel have very similar thermal expansion coefficients and, therefore, good adhesion is conserved between the two. Due to the excellent mechanical properties, low cost, and workability, reinforced concrete is shaping buildings and urban landscapes all over the globe [1].

Concrete is also very durable. Some buildings from the Roman Empire still exist 2000 years after construction. However, typical structures have a much more limited lifetime due to different degradation processes [2], which can be divided into physical (like those caused by freeze-thaw cycles or by fire), mechanical (due to erosion, abrasion, or impact), chemical (reactions with acids, ammonium, sulfate, CO_2_, or chloride), biological, and structural (e.g., overloading, cyclic loads, and soil settlement).

A major cause of the early degradation of reinforced concrete structures is the corrosion of steel rebars [2,3,4]. In general, the high alkalinity of concrete is an excellent environment for the passivity of steel. However, the ingress of aggressive agents like CO_2_ from the atmosphere or chloride ions from maritime environments and from deicing activity creates conditions for passivity breakdown. Carbon dioxide leads to the carbonation of concrete, which is accompanied by a decrease in pH. When the front of low pH reaches the steel surface, the passivity is lost and uniform corrosion starts. Chloride ions, on the other hand, can disrupt the passive layer even at high pH, if a threshold concentration is surpassed [5]. In these conditions, the corrosion is localized in the form of pitting, crevice, or stress corrosion cracking. The iron corrosion products are more voluminous than steel, which creates expansive stresses, leading to cracking at first and spallation of the concrete cover at the end. After that, steel becomes directly exposed to the atmospheric environment, and corrosion proceeds at a much faster rate.

Since the corrosion of steel rebars dramatically limits the service life of reinforced concrete, many forms of corrosion control are being explored [2,3,4,6,7], including the use of stainless steel or galvanized steel rebars, application of epoxy coating on steel rebars, cathodic protection, and addition of corrosion inhibitors to concrete (e.g., calcium nitrite, sodium benzoate, chromates, phosphates, polyphosphates, silicates, polycarboxylic acids, fatty acids emulsions, and alkanolamines). Corrosion can also be delayed by painting the surface of the concrete structure or using a thicker layer of concrete, separating the rebars and the environment. Less porous concrete (lower water/cement ratio), high quality cement, and the use of water and aggregates without soluble salts also contribute to extending the durability of the structure.

The direct addition of corrosion inhibitors to concrete may affect the curing process or the mechanical properties of the hardened material. Because of this possibility, the encapsulation of inhibitors in nano- or micro-reservoirs to be released only when needed (either with the onset of corrosion or in the presence of aggressive species) is a line of investigation worth pursuing.

Layered double hydroxides (LDHs) are one example of such nanostructured reservoirs. The LDH structure resembles the layered structure of Mg(OH)_2_ (brucite), with trivalent cations replacing some divalent Mg^2+^ cations [8,9]. The resulting excess of positive charge is compensated by anions between the metal hydroxide layers. Water is also in the interlayer space and helps to maintain the integrity of the structure by forming hydrogen bonds. The general formula of LDH is [M^2+^_1−x_M^3+^_x_(OH)_2_]^+^[A^n−^]_x/n_·yH_2_O with M^2+^ = Mg^2+^, Ca^2+^, Zn^2+^, Ni^2+^, or Mn^2+^, M^3+^ = Al^3+^, Fe^3+^, Cr^3+^, or Ga^3+^, and A^n−^ = CO_3_^2−^, OH^−^, Cl^−^, NO_2_^−^, or NO_3_^−^, to name just a few. Hydrotalcite is a naturally-occurring LDH with the chemical formula Mg_6_Al_2_CO_3_(OH)_16_·4H_2_O, where Al^3+^ is the trivalent cation, and CO_3_^2−^ is the anion in the interlayer space. The intercalated anions can be exchanged with other anions available in the environment, a property that is exploited in different areas, from catalysis to medicine [8,9,10,11]. The application to corrosion control relies on the LDH capacity to capture aggressive anions from the environment (e.g., chloride ions) [12], while releasing anionic inhibiting species to maintain charge neutrality [13]. LDHs can be used as additives incorporated into organic coatings [14] or as a conversion film directly grown on the metallic substrates [15].

Studies of the action of LDH in concrete are also available in the literature. In 2003, a paper was published reporting the use of a synthetic nitrite type of hydrocalumite (CaAl-LDH) to counteract the chloride penetration in concrete through a so called suppressing salt injury (SSI) method [16]. The LDH was added to the concrete as chloride absorbent. The process involved the ionic exchange with nitrite ion (corrosion inhibitor) that was released to the environment. Still today, this remains the main concept behind the anticorrosion action of LDH in reinforced concrete. Concrete with this LDH was used for repairing a viaduct pier and a platform abutment. One year later, in 2004, a paper reported the use of nitrobenzoic acid, naphthalene-2, 6-disulfonic acid, and naphthalene-2 sulfonic acid salts, intercalated in CaAl-LDH, with the aim of controlling the kinetics of cement hydration by programming the temporal release of those compounds [17]. The use of LDH to control the cement hydration kinetics has been studied by several groups [18,19,20,21]. However, most of the work with LDH in concrete is dedicated to the mitigation of steel rebars corrosion, either in chloride media [22,23,24,25,26,27,28,29,30,31,32,33,34,35,36,37,38,39,40] or carbonated concrete [32,41,42,43,44,45,46,47,48]. Many studies have been done in simulated concrete pore solution [35,38,43,46], just a few in mortar [31,43] and only one in concrete [16]. Often, the workability and mechanical properties of mortar and concrete with LDH are also characterized [18,19,20,21,22,28,37,41]. Recent reports describe the use of LDH as an anti-corrosive pigment in epoxy coatings for steel rebars [49] and a field evaluation of LDH effect on the aging resistance of asphalt concrete after four years of road service [50].

In the above-mentioned studies, the intercalated anions were nitrite (corrosion inhibitor), nitrate, carbonate, and chloride. The most commonly studied LDH is by far MgAl-LDH [20,21,22,24,27,32,35,37,38,39,41,42,43,44,45,46,47,49,50], followed by CaAl [16,17,18,26,32,33,34,40], hydrotalcite [23,29,36], and modified hydrotalcite [25,28,31]. ZnAl-LDH is a new composition that has been studied in only two papers [51,52]. One has analyzed the LDH ability to capture chloride and release nitrite in NaCl solutions of different concentrations and characterized the corrosion protection in saturated Ca(OH)_2_ solution after the addition of LDH. The other paper has presented three ZnAl-phthalate LDHs prepared by different methods and compared their effect on the corrosion resistance of steel in saturated Ca(OH)_2_ solution adjusted to pH = 11.

This work aims at extending the current knowledge on the action of ZnAl-LDH in cementitious materials by investigating the behavior of the LDH in aqueous solution, in cement paste, and in mortar. The analyzed parameters were the stability of LDH in aqueous solutions of different pH, the capacity of ZnAl-LDH for capturing chloride ions in solutions of different chloride concentration and pH, the effect on the corrosion of steel in aqueous solution, the influence on the curing of cement paste, and the impact on the corrosion of steel rebar embedded in mortar.

## 2. Materials and Methods

### 2.1. Synthesis of ZnAl-NO_3_ and ZnAl-NO_2_ LDHs

The LDH materials studied in this work were produced by Smallmatek, Lda (Aveiro, Portugal) and prepared according to their own production procedures. Briefly, 0.5 M Zn(NO_3_)_2_·6H_2_O and 0.25 M Al(NO_3_)_3_·9H_2_O were slowly added to 1.5 M NaNO_3_ or NaNO_2_ solution under vigorous stirring at room temperature. During this reaction, the pH was kept constant (pH = 10 ± 0.5) by the addition of 2 M NaOH solution. The production process was carried out in a custom-made stainless-steel pilot-scale reactor (BTL-Indústrias Metalúrgicas, S.A., Oliveira de Azeméis, Portugal) equipped with a PID (proportional–integral–derivative) controller, which allowed the automatic control and correction of important process parameters (e.g., pH and temperature), and peristaltic pumps for precise addition of chemicals. The obtained slurry was washed with deionized water and filtered under reduced pressure, followed by drying using an industrial spray dryer to guarantee uniform and fine powders. The powders were finally separated in different size fractions using a vibratory sieve shaker (Retsch, Haan, Germany).

### 2.2. Structure and Morphology of the ZnAl LDH

Scanning electron microscopy (SEM) was performed on Hitachi (Tokyo, Japan) S-4100 field emission electron microscope with an accelerating voltage of 25 kV. X-ray diffractograms (XRD) were acquired using a PANalytical X’Pert MPD PRO diffractometer (Almelo, The Netherlands) with Bragg-Brentano geometry, Ni-filtered CuKα radiation, PIXcel1D detector, and step 0.026. The exposition corresponded to about 2 s per step over the angular range between 3 and 70. The particle size distribution of the different LDH batches was measured on a LS230 particle sizer (Coulter Corporation, Miami, FL, USA). Fourier transform infrared spectroscopy (FT-IR) spectra were collected in a Spectrum Two spectrometer (PerkinElmer Inc., Waltham, MA, USA) with a UATR TWO unit (Diamond), 64 scans, 4 cm^−1^ resolution, in a wavelength range of 400–4000 cm^−1^.

### 2.3. Stability of ZnAl LDH in Aqueous Solution

The stability of LDH powders in water in the pH range from 1 to 14 (adjusted with HNO_3_ or KOH) was evaluated by placing 1 g of LDH in 50 mL of each solution (in intervals of 1 pH unit) for one month. After this period, the remaining powder was weighted (after washing in distilled water and drying).

### 2.4. Ion Exchange in Aqueous Solution

The capacity of the LDHs for capturing chloride ions was assessed by potentiometry, using a chloride ion-selective electrode (DX235-Cl from Mettler Toledo, Columbus, OH, USA) with a mercury/mercurous sulfate reference electrode (REF621, Radiometer Analytical SAS, Lyon, France) connected to a SevenMulti meter from Mettler Toledo, Columbus, OH, USA. Aqueous NaCl solutions of various concentrations and pH were continuously analyzed with the Cl^−^ sensor. After a time for stable reading, 1 g of LDH was added to 50 mL solution under stirring, while the sensor kept measuring.

### 2.5. Effect of ZnAl LDH on the Corrosion of Steel in Aqueous Solutions

Cross-sections of steel rebars with one side electrically connected to the copper wire were mounted in epoxy resin. The other side was ground down to SiC 4000 grade and covered with insulating tape (Electroplating Tape 470M, 3M, Saint Paul, MN, USA) with a circular opening of 0.24 cm^2^, to provide a constant area of analysis for all measurements and to limit the risk of crevice corrosion in the experiments. The corrosion was analyzed by electrochemical impedance spectroscopy (EIS) using IVIUM CompactStat (Ivium Technologies, Eindhoven, The Netherlands) or Autolab PGSTAT204 (Metrohm Autolab, Utrecht, The Netherlands) potentiostats, in a tree-electrode arrangement, with the steel sample as working electrode, a saturated calomel electrode (SCE) as a reference, and a platinum counter electrode. The samples were immersed in 0.05 M NaCl (pH = 5.8) or 0.05 M NaCl + 0.1 M KOH (pH = 13), with and without LDH (0.5% mass/volume), and the corrosion monitored over time, with EIS spectra acquired at open circuit potential (OCP) with a sine wave perturbation of 12 mV rms, from 10^5^ to 10^−3^ Hz with 10 points per decade with logarithmic distribution.

### 2.6. Cement Pastes and Mortars Preparation

Cement pastes with different LDH additions were prepared with CEM II/B-L 32.5 N cement and distilled water (0.5 water/cement ratio). The specimens were cubes with 2.5 cm edges. The same cement and water, together with 0–2 mm size siliceous sand, were used to prepare mortars (14.5 wt.% cement + 13 wt.% water + 72.5 wt.% sand). The water/cement ratio was 0.9, a value selected to warrant high porosity and faster corrosion of steel. The quantity of LDH added to the mortars was 0.3% of their total mass (corresponding to 2% of the mass of cement). The size of the mortars was 7 × 4 × 4 cm^3^, and a non-corrugated steel bar of 8 mm diameter was placed in the middle (nominal composition of steel (%): Fe, 98.08; C, 0.20; Mn, 0.70; Cu, 0.40; Si, 0.20; Cr, 0.20; Ni, 0.10; S, 0.05; P, 0.02; Mo, 0.02; V, 0.02; N, 0.01). Prior to the embedment in the mortars, the steel bars were etched in HCl aqueous solution (50 vol.%), abraded with SiC paper down to 1200 grade, and passivated in 0.1 M KOH solution for 3 h. The area of steel bar inside the mortar was 12.25 cm^2^. After 24 h in the mold, the mortars were left curing for 8 days immersed in water. This time was sufficient for mechanical integrity and short enough to warrant fast degradation.

### 2.7. Chloride Sensors inside Mortars

Ag|AgCl potentiometric sensors were placed inside mortars for the direct measurement of chloride ingress. The sensors were produced on 1 mm diameter Ag wires embedded in epoxy. The silver chloride layer was formed at the polished Ag surface by applying a constant current of 2 mA/cm^2^ in 0.1 M HCl for 30 min. The sensors were placed at 0.5, 1, and 1.5 cm from the surface of the mortar samples. After 28 days of curing the mortars were immersed in 3.5% NaCl solution. The potential was measured with respect to a saturated calomel electrode located in the external NaCl solution, using a CompactStat potentiostat connected to a peripheral differential amplifier (both from Ivium Technologies, Eindhoven, The Netherlands) for simultaneous measurements.

### 2.8. Impact of ZnAl LDH on the Corrosion of Steel Rebars in Mortars

Mortars with steel bars were immersed in 3.5% NaCl solution, and impedance measurements were performed in the same conditions described in 2.5, except for the smaller number of points per decade (5 points) to save measurement time due to the larger number of samples to test.

## 3. Results and Discussion

### 3.1. Structure and Morphology of the Synthesized ZnAl LDHs

**Figure 1:**
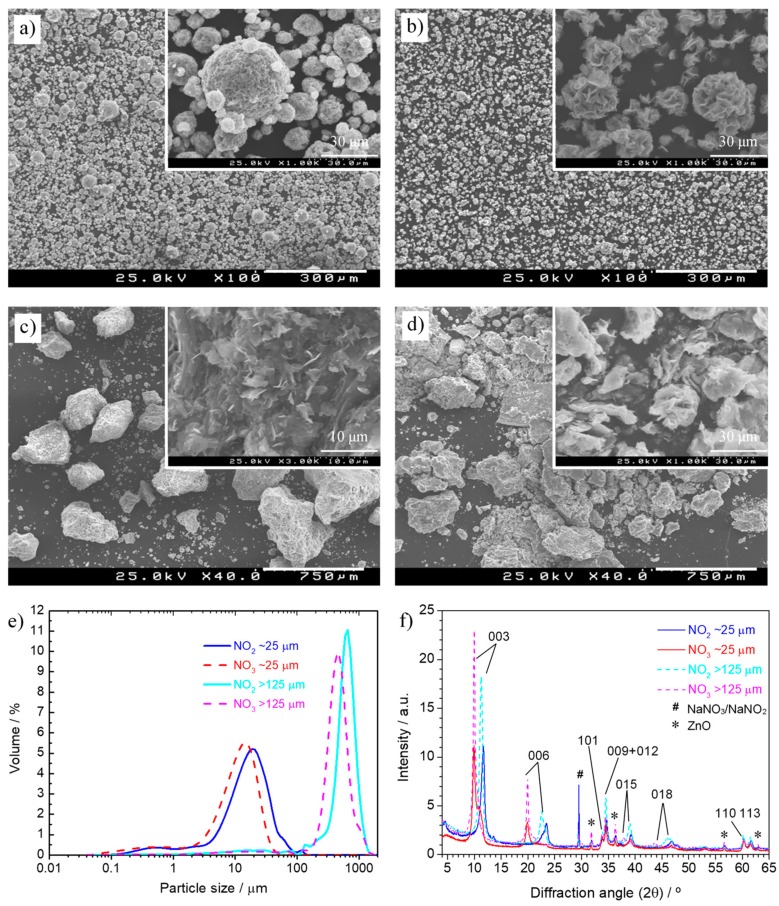
Scanning electron microscopy (SEM) images of (**a**) ZnAl-NO_2_ with mean particle size ~25 µm, (**b**) ZnAl-NO_3_ with mean particle size ~25 µm, (**c**) ZnAl-NO_2_ with particle size >125 µm, (**d**) ZnAl-NO_3_ with mean particle size >125 µm; (**e**) particle size distribution of the layered double hydroxide (LDH) powders; (**f**) XRD diffractograms of the four LDH powders.

ZnAl-NO_3_ and ZnAl-NO_2_ powders of different particle sizes were studied in this work. Figure 1a–d show SEM images of the different batches. In all cases, it was possible to identify the lamellar shape typical of LDH materials. The particle size distribution is presented in Figure 1e and reveals two batches of particles in the range of 25 µm (slightly smaller for ZnAl-NO_3_), both with a small fraction around 0.5 µm, and two batches of particles above 125 µm, with mean values, centered on 650 µm for ZnAl-NO_2_ and 450 µm for ZnAl-NO_3_. Figure 1f depicts the XRD patterns of the LDH powders. The X-ray diffraction data showed the same LDH phase regardless of the particle size.

The diffractograms of ZnAl-NO_3_ of different particle size practically overlapped and showed the characteristic (003), (006), (110), and (113) reflections occurring, respectively, at 10°, 20°, 60.5°, and 61.5°, confirming the formation of LDH intercalated with NO_3_^−^ [13]. On ZnAl-NO_2_, the peaks shifted to higher angles, which was indicative of the intercalation of smaller anions. The (003), (006), (110), and (113) reflections now occurred around, respectively, 11.6°, 23°, 60.2°, and 61.5°, values similar to those found with a similar ZnAl-NO_2_ [51] and other LDHs with NO_2_^–^ in the interlayer space [31,35,43].

**Figure 2:**
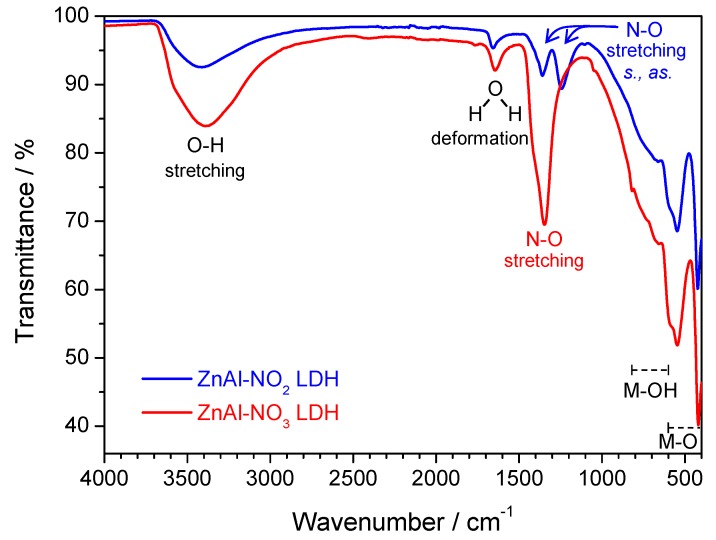
FTIR spectra of ZnAl-NO_3_ and ZnAl-NO_2_.

FTIR analysis was used to further confirm the presence of nitrate and nitrite ions in the LDH structures—Figure 2. The characteristic bands of LDH were visible in both spectra, namely, a broad band around 3400 cm^−1^ corresponding to stretching vibrations of the hydroxyl groups of both layer hydroxide moieties and interlayer water, a band at 1660 cm^−1^ due to the deformation vibration mode of OH bonds in water molecules, and bands at 1000–600 cm^−1^ assigned to the metal-hydroxide group connection (M-OH) and at 600–400 cm^−1^ ascribed to the metal-oxygen link (M-O) [53]. The main differences between ZnAl-NO_3_ and ZnAl-NO_2_ relied on the N–O vibration modes, being a single stretching band at 1346 cm^−1^ for nitrate and two bands at 1358 and 1244 cm^−1^ for nitrite, related to the symmetric and asymmetric stretching.

### 3.2. Stability of ZnAl LDH in Aqueous Solution

**Figure 3:**
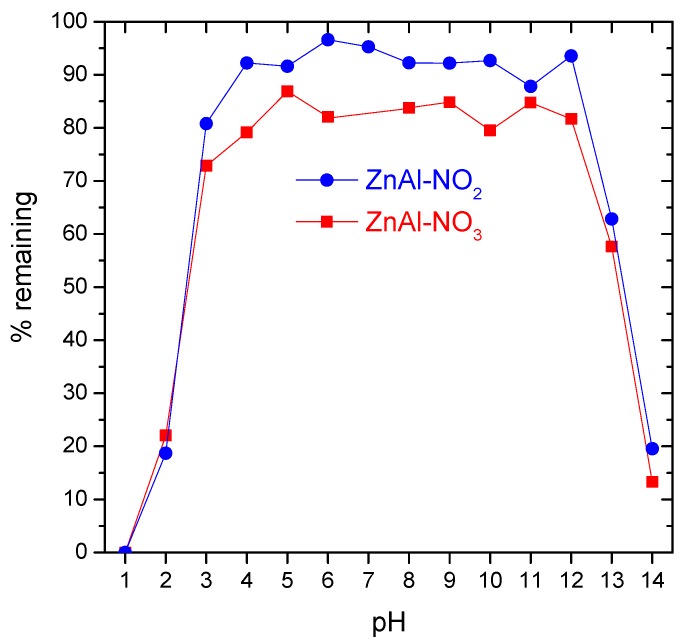
Mass (%) of undissolved LDH powder after 1 month of immersion in water in the pH range 1 to 14.

The stability of this type of material in aqueous solution has great practical importance. In order to encompass all possible applications, the stability of the LDHs was studied in the full pH range. Naturally, for applications in concrete, it is the alkaline region that matters because the typical pH of cement paste, mortar, or concrete ranges from 12.5 (fresh) to 13.5 (cured) [2,3,4]. Figure 3 shows the amount of LDH that remained after 1 month in contact with water in the pH range from 1 to 14. The results indicated that ZnAl-LDH was unstable at extreme pH values, either in acidic or alkaline conditions. It dissolved completely in pH = 1, while in pH = 2 only 20% of solid remained. In a solution with pH = 13, around 40% of the sample was dissolved, whereas in a solution with pH = 14 just 20% of powder remained. This dissolution in the high alkaline region suggested that the same was likely to occur in cementitious environments, which could be critical for the application of these LDHs in concrete. In the pH range from 3 to 12, the LDH was less affected, though around 10% of ZnAl-NO_2_ and 20% of ZnAl-NO_3_ tended to dissolve, according to Figure 3. The dissolution at mild pH values has been discussed in a previous work by Galvão et al. [54].

### 3.3. The Capacity of ZnAl LDH for Capturing Chloride Ions

**Figure 4:**
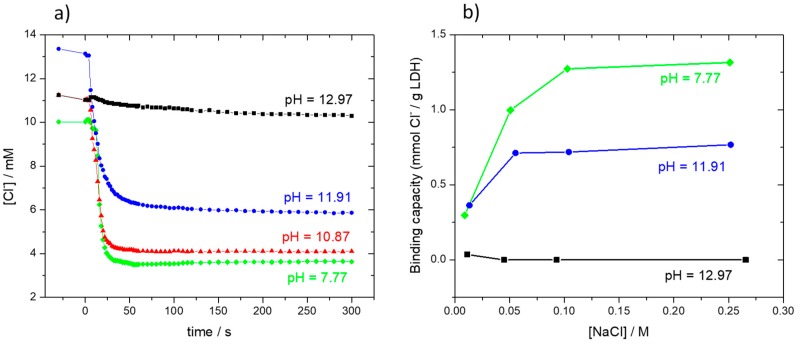
(**a**) Decrease of chloride concentration of 0.01 M NaCl solution at different pH after the addition of ZnAl-NO_2_ (solution volume = 50 mL, 1 g of LDH added at time t = 0); (**b**) Chloride binding capacity of ZnAl-NO_2_ at different pH and chloride concentrations.

One of the key properties for the use of LDHs in corrosion control is the capacity to capture aggressive species, namely, chloride ions. Figure 4a shows the decrease in chloride concentration of NaCl solutions after the addition of LDH. The process was fast, occurring in the first minutes after the addition of the powder to the solution, and led to a significant capture of chloride ions. However, the ability to capture chloride decreased with the increase in pH and was completely lost at pH~13. The decrease in the ability to capture chloride ions at high pH was attributed to two factors. The first, based on Figure 3, was the partial dissolution of the LDH at high pH, leading to a smaller amount of material with capture ability. The second was the higher tendency for the ZnAl-LDH to exchange with OH^–^ ions instead of Cl^–^ ions, an evidence based on the results of Figure 4 and a behavior similar to other LDHs [55]. The results obtained in this work point to the following order of anion selectivity: NO_3_^−^ < NO_2_^−^ < Cl^–^ < OH^−^. Ions to the right enter the LDH preferentially, and, once inside, they are hardly displaced by those on the left.

The effect of both chloride concentration and pH on the amount of chloride captured by ZnAl-LDH can be easily perceived in Figure 4b, which was determined using ZnAl-NO_2_ LDH. This figure presents the chloride binding capacity *B_c_*, defined as the amount of chloride that is bound (captured) by a given mass of LDH, and calculated using the following equation [46,56]:

(1) $$B_{c}=\frac{V_{sol}\;\left(C_{0}-C_{e}\right)\;}{m_{LDH}}$$

where *B_c_* is the binding capacity (moles_Cl_/g_LDH_), *V_sol_* is the volume of solution (dm^3^), *m_LDH_* is the mass of LDH added to the solution (g), and *C*_0_ and *C_e_* are, respectively, the initial and equilibrium chloride concentrations (mol dm^−3^). The binding capacity increased with chloride concentration until a value where it remained constant. The maximum value, *B_c_* = 1.25 mmol_Cl_/*g*_LDH_, was measured in near-neutral solution and for [Cl^−^] > 0.1 M. In agreement with Figure 4a, the binding capacity was strongly dependent on the pH, decreasing with it, and becoming zero at pH~13. This result is of utmost importance for applications in concrete.

### 3.4. Effect of ZnAl LDH on the Corrosion of Steel in Aqueous Solution

**Figure 5:**
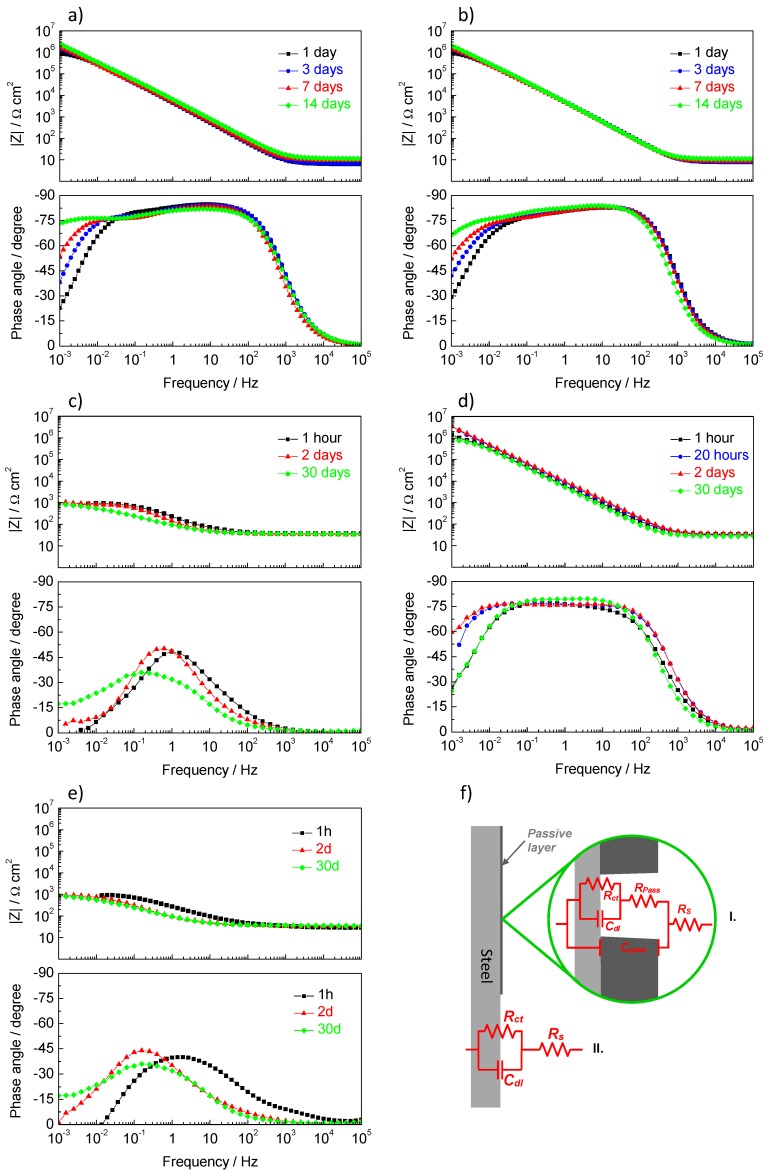
EIS spectra of steel obtained during immersion in (**a**) 0.05 M NaCl (pH = 13), (**b**) 0.05 M NaCl (pH = 13) + 0.5% ZnAl-NO_2_, (**c**) 0.05 M NaCl (pH~6), (**d**) 0.05 M NaCl + 0.5% ZnAl-NO_2_, (**e**) 0.05 M NaCl + 0.5% ZnAl-NO_3_, (**f**) equivalent electric circuits used for describing the impedance response.

To simulate the ability of ZnAl-LDH to control the corrosion of steel in the concrete environment, steel samples were immersed in 0.05 M NaCl solutions with pH = 13 (~0.1 M KOH) with and without 0.5 wt.% ZnAl-NO_2_. The NaCl concentration and the amount of LDH in solution were chosen to be similar to past works [13]. Bode plots of the impedance of the steel measured during the time of immersion are presented in Figure 5a (control, without LDH) and Figure 5b (with 0.5% LDH). Similar EIS spectra were obtained for these systems, namely, high impedance values at intermediate and low frequencies, which tended to increase with time of immersion. Moreover, two time constants were detected, one at higher frequencies, attributed to the passive film formed under alkaline conditions, and another at low frequencies (~10^−2^ Hz) ascribed to small active areas of steel coincident with pores and defects in the passive film. With time, these pores and defects decreased in size and number (due to the passivating effect of the alkaline environment and the presence of inhibitors), which explains the increase in impedance with time.

Since the reference system presented already high impedance, the effect of the LDH could not be easily identified. The effect became evident in 0.05 M NaCl solution without pH adjustment (pH~6). This slightly acidic pH was due to the hydrolysis of dissolved CO_2_ from the atmosphere [57,58]. The corrosion of steel, in this solution, was fast with impedance at low frequencies around 1 kΩ cm^2^ (Figure 5c). The addition of 0.5% ZnAl-NO_2_ LDH led to passivation of the steel sample, with a considerable increase in impedance and the typical capacitive behavior of passive metals (Figure 5d). Indeed, at the end of the test (1 month), the surface was still intact and shiny. Two effects might have contributed to this result: the capture of chloride ions from the solution and the release of nitrite ions in an amount enough to act as a corrosion inhibitor. The addition of a similar quantity of ZnAl-NO_3_ to 0.05 M NaCl did not produce any significant change, being the impedance response (Figure 5e) similar to that of the blank solution (Figure 5c). This means that the amount of captured chloride was not that significant compared to the quantity of chloride ions still remaining in solution. These results lead to the conclusion that the key factor for the action of LDH in the present conditions is the release of nitrite ions to the solution.

The impedance responses were numerically fitted with the Zview software (Version 3.5g, Scribner Associates, Southern Pines, NC, USA) using the equivalent electric circuits of Figure 5f. The circuit I served as an analog of the passive surface with the solution resistance (*R_s_*), the capacitance of the passive film (*C_pass_*), the resistance of the solution inside the pores and defects of the passive film (*R_pass_*), double layer capacitance (*C_dl_*), and charge transfer resistance (*R_ct_*). *C_dl_* and *R_ct_* occurred at the bottom of the pores and defects of the passive film, in the points where the metal was directly exposed to the solution. Circuit II was the analog of the active steel surface with just three elements, *R_s_*, *C_dl_,* and *R_ct_*. In some cases, this simple circuit can be more complicated, with the appearance of the response of a deposit of corrosion products at higher frequencies and/or the manifestation of diffusion control at lower frequencies.

In the fitting procedure, constant phase elements (CPE) were used instead of capacitances, to account for the non-ideal capacitive behavior of the surface arising from the heterogeneities and uneven reactivity on the electrode surface [59]. The CPE values were converted to capacitances using the equation proposed by Brug et al. [60],

(2) $$C=\sqrt[{n}]{Y_{0}\;}{\left(\frac{1}{R_{1}}+\frac{1}{R_{2}}\right)}^{\frac{n-1}{n}}$$

where *Y*_0_ and *n* are, respectively, the frequency-independent admittance and the power of the CPE, and *R*_1_ and *R*_2_ are the resistances in series and in parallel with the CPE, respectively.

The parameters from the fitting analysis and application of Equation (2) are presented in Table 1. The evolution of the parameters was in line with the qualitative description presented above. At high pH, the impedance of steel was high and increased with time, particularly by the increase of *R_pass_* and *R_ct_*. The capacitances *C_pass_* and *C_dl_* did not change significantly. The effect of nitrite at high pH was manifested mainly by a slightly higher *R_ct_* and a lower *C_dl_* compared to the blank solution. The effect of nitrite was visible in near-neutral pH, where the impedance was 3 orders of magnitude higher than the non-inhibited NaCl solution, mainly due to the high *R_ct_*, which was in the same order as the samples in alkaline solution. The parameter values of steel in NaCl solution with or without ZnAl-NO_3_ were similar, with high capacitances and low resistances, particularly *R_ct_*. The fitting in the case of ZnAl-NO_3_ needed to consider a time constant at higher frequencies attributed to a surface layer, either a reminiscence of the weak native passive film or the formation of a layer of corrosion products. These results showed the ability of ZnAl-NO_2_ to significantly increase the corrosion resistance of steel. No effect was found for ZnAl-NO_3_.

### 3.5. Influence of ZnAl LDH on the Hardening of Cement Paste

**Figure 6:**
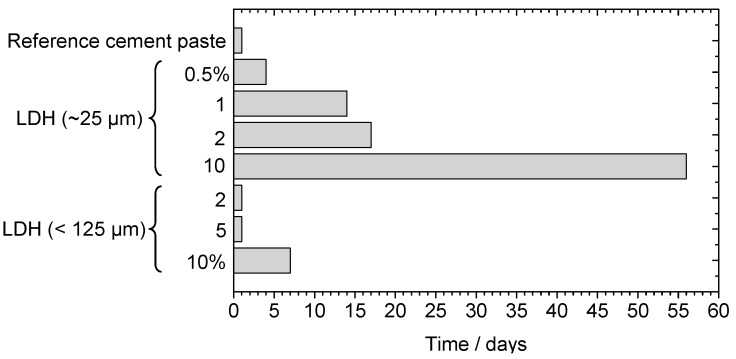
Influence of LDH particle size on the curing time of cement paste (% of LDH with respect to the mass of cement).

After characterizing the LDH powders in aqueous solution, and before the incorporation in a mortar, LDHs were added to cement paste to verify the influence on the curing time. The variables analyzed were the type of LDH (ZnAl-NO_2_ or ZnAl-NO_3_), the amount added, and the LDH particle size used. The parameter under analysis was the time needed to demold the specimens prior to the curing stage in water—Figure 6. A reference sample was typically demolded in 24 h. Upon the addition of ZnAl-LDH, an increase in demolding time was observed. Clearly, the particle size had influence. Too many days were necessary for hardening in the case of LDH particles with a size of around 25 μm (ZnAl-NO_2_ or ZnAl-NO_3_). Furthermore, the hardening time increased significantly as the amount of LDH increased. On the other hand, using LDH particles of larger size (>125 μm) allowed for demolding times similar to the reference samples, except for the highest amount tested (10%), where the specimens took 7 days to harden before demolding. No differences were found between ZnAl-NO_2_ and ZnAl-NO_3_.

The explanation for the increase in hardening time was attributed to the partial dissolution of LDH, induced by the high pH of the cement paste, which released zinc ions (Zn(OH)_3_^−^ and Zn(OH)_4_^2−^ at high pH) that are known to retard cement hydration [61,62,63,64]. In the cement pore solution, the presence of zinc ions leads to the formation of a new phase, Ca(Zn(OH)_3_)_2_·2H_2_O [62,63,64]. While zinc ions existed in solution, this phase was formed, and the concentrations of OH^−^ and Ca^2+^ were kept low, preventing the hydration reactions. Only after the depletion of zinc ions, the hydration was able to proceed at a normal rate. In addition, Asavapisit et al. [63] referred that the new Ca(Zn(OH)_3_)_2_·2H_2_O phase could be deposited over the cement particles, forming a membrane with low permeability, preventing the particles from further hydration reactions.

### 3.6. Impact of ZnAl LDH on the Corrosion of Steel Rebars in Mortars

As the last step prior to testing in concrete, experiments were conducted in mortars with sensors to monitor the chloride ingress or with embedded steel rebar for corrosion testing.

#### 3.6.1. Chloride Sensors inside Mortar

**Figure 7:**
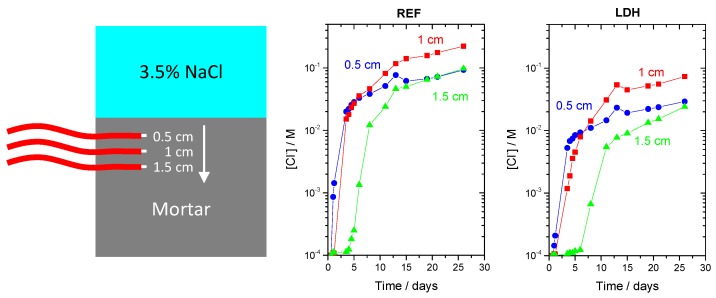
Scheme of mortar with sensors and chloride profiles inside mortar without (REF) and with ZnAl-NO_2_.

Figure 7 shows a schematic representation of a mortar sample with embedded sensors immersed in 3.5% NaCl aqueous solution and the chloride concentration profiles in a reference mortar (REF) and in a mortar with 0.3% ZnAl-NO_2_ (2% with respect to cement). The chloride concentration inside the mortar was calculated based on the potential readings by the sensor and applying a calibration curve obtained with NaCl solutions at pH = 13. The results showed that chloride was detected first by the sensors closer to the surface and in the reference sample. The sample containing LDH presented lower values of chloride for the same depth and time of exposure compared to the reference sample. This means that the addition of LDH was able to slow down the chloride transport in the mortar, in spite of the small amount used (0.3% of total mass) and the high porosity of the mortar. An interesting observation was the peak of chloride concentration occurring not at the surface but a few millimeters inside the mortar. This has been observed many times [65,66,67] and explained by a difference in composition of the concrete skin (the region closest to the surface) due to the contact with mold walls, segregation of aggregates, and environmental actions, inducing a gradient of moisture along the cover depth [65]. It has also been proposed that surface chemical reactions with the environment change the composition of the outer layer (concrete skin) of the concrete [67].

#### 3.6.2. Electrochemical Impedance Spectroscopy

**Figure 8:**
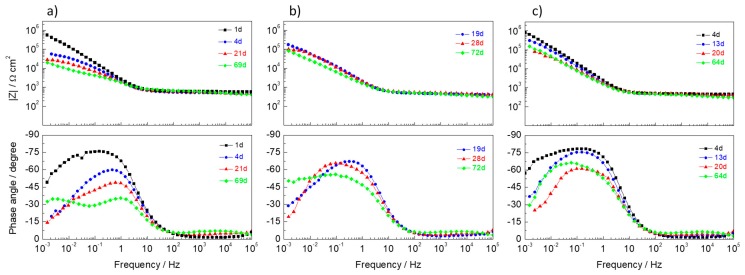
Impedance response of mortars with steel bar immersed in 3.5% NaCl: (**a**) Mortar without LDH (reference), (**b**) mortar with 0.3% ZnAl-NO_3_ (2% with respect to cement), (**c**) mortar with 0.3% ZnAl-NO_2_ (2% with respect to cement).

**Figure 9:**
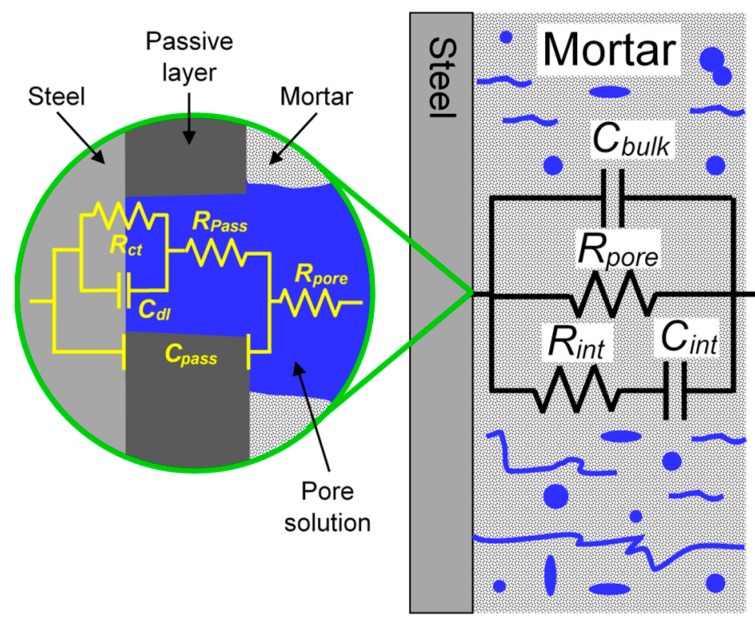
The equivalent electric circuit used for describing the impedance of mortar with a steel bar.

The final tests were performed in steel rebars inside mortars and immersed in 3.5% NaCl. The samples were monitored by EIS during the immersion time. Bode plots are presented in Figure 8, and Table 2 shows the parameters obtained by the numerical fitting analysis with capacitances calculated by Equation (2). The equivalent electric circuit used was similar to those shown in Figure 5f, except that *R_s_* is now called *R_pore_* and stands for the resistance of the testing solution in series with the resistance of the solution in the pores structure inside the mortar. Figure 9 depicts the circuit in more detail. The picture showed separately the response in the mortar and the response at the steel interface. The circuit for the response in the mortar was the one proposed for hardened Portland cement paste [68], where *C_bulk_* accounts for the dielectric capacitance associated with the solid phase, *R_pore_* is related to ionic motion in pores, crossing the bulk material from the solution side to the steel surface side, and *C_int_* and *R_int_* are used to account for the ionic motion in regions internal to the sample, including occluded pores [68]. Due to the high porosity of the present mortars, the response was believed to be dominated by *R_pore_*. Then, at the steel interface, and because of the high alkalinity of the mortar, the circuit of a passive metal with defects (like in Figure 5f) was used. The circuit was, in fact, similar to what could be found in the literature [69].

The spectra showed two distinct regions, one at frequencies above ~10 Hz with the resistive response of *R_pore_* and another below ~10 Hz, with the response of the steel surface, with one or two time constants. The impedance at high frequencies was relatively low (~500 Ω cm^2^), not changing significantly with the time of immersion. These values reflected the high porosity of the mortars and the absence of any effect of LDH (at least in the amount added) in counteracting porosity.

Regarding the response of steel, the reference sample (Figure 8a) started with a capacitive behavior due to the initial passive state of the rebar, but a noticeable decrease in impedance occurred in just a few days together with the appearance of a second time constant at lower frequencies, revealing the onset of localized corrosion as a result of the fast entrance of chloride ions through the porous mortar. The samples with LDH (Figure 8b,c) were able to keep the impedance at higher values. Even when a second time constant was detected at low frequencies, impedance remained higher compared to the reference mortar. After 2 months of immersion, the *R_ct_* of samples with LDH were 1−2 × 10^5^ Ω cm^2^, about 2–4 times higher than the reference. Due to the smaller amount of chloride ions inside the mortar compared to the study in solution (Section 3.4), the LDHs seemed to be playing a role in capturing chloride ions, thus increasing the corrosion resistance of the steel bar (fewer chloride ions to attack the passive layer—higher *R_pass_*—and less area under corrosion—higher *R_ct_*).

These are the preliminary results of experiments performed with a limited number of samples (2–3 of each system) and a small addition of LDH to the mortars (0.3% of total mass). A more comprehensive study is presently being conducted.

### 3.7. Final Remarks

This work shows that ZnAl-NO_2_ can be used to control the corrosion of steel in aqueous solution. However, it also shows that at the high pH of concrete, the ZnAl-LDHs partially dissolves, and OH^–^ is exchanged preferentially to chloride. These two facts have a direct impact on the use of these LDHs to control the corrosion of steel in mortar and concrete but are often neglected in studies in the field.

The partial dissolution at high pH and the preferential exchange by OH^–^ suggest that these LDH might not be adequate for use in cementitious materials. Contrariwise, some works in the literature report higher corrosion resistance and durability of mortar and concrete with LDH [16,31,43]. The results in this work with sensors and steel bars embedded in mortars showed lower chloride penetration and higher corrosion resistance on samples with these LDH. At the moment, it is not possible to conciliate the absence of LDH effect suggested by the results in a solution with the positive effects observed in mortars. These effects might not come from the LDH structure but from chemical reactions with the ions dissolved from the LDH. It appears that the LDH chemistry alone cannot explain the action inside the concrete, and other reactions might take place, leading to the observed improvement in the performance. This is currently under investigation and will be reported in due time.

## 4. Conclusions

The results of this work confirmed the ability of ZnAl-LDH to capture chloride ions from solution and release nitrate/nitrite ions in their place. The ion exchange reaction was very fast, occurring in just a few minutes. This process was pH-dependent and stopped at high pH due to the partial dissolution of LDH and the preferential exchange of OH^–^ ions. The higher corrosion resistance of steel was observed when ZnAl-NO_2_ was added to the aqueous solution. ZnAl-NO_3_ did not produce any noticeable effect.

The LDH powders dissolved partially at pH > 12. Particles around ~25 μm delayed the curing of cement, while larger agglomerates (>125 μm) allowed the same hardening time as the reference mortar (without LDH). The delay was attributed to the dissolution of Zn^2+^ ions from the LDH and their interference with the cement hydration reaction.

The partial dissolution and preferential capture of OH^−^ at high pH can compromise the use of these LDHs in concrete. However, preliminary results showed lower penetration of chloride ions and higher resistance to corrosion of steel rebars. A more comprehensive study is currently underway.

## Figures and Tables

**Table 1:** Parameters determined by numerical fitting of the EIS spectra shown in Figure 5 with capacitances calculated using Equation (2).

pH	Composition	Time	*R_s_* (Ω cm^2^)	*Y_0,pass_* (F cm^−2^ s^n−1^)	n_pass_	*C_pass_* (μF cm^−2^)	*R_pass_* (Ω cm^2^)	*Y_0,dl_* (F cm^−2^ s^n−1^)	*n_dl_*	*C_dl_* (μF cm^−2^)	*R_ct_* (Ω cm^2^)	*Χ^2^*/10^−4^
13	Blank	1d	6.7	3.07 × 10*^−^*^5^	0.968	23.1	50.53	6.86 × 10*^−^*^6^	0.701	0.23	1.09 × 10^6^	0.83
		3d	6.9	3.55 × 10*^−^*^5^	0.946	22.1	80200	9.44 × 10*^−^*^6^	0.794	8.69	2.00 × 10^6^	0.37
		7d	9.8	3.45 × 10*^−^*^5^	0.917	16.7	81882	1.14 × 10*^−^*^5^	0.793	11.1	3.87 × 10^6^	0.36
		14d	11.7	2.76 × 10*^−^*^5^	0.921	13.8	220140	5.95 × 10*^−^*^6^	0.740	6.52	4.27 × 10^7^	0.27
	0.5% LDH-NO_2_	1d	8.0	3.17 × 10*^−^*^5^	0.945	19.6	19353	8.37 × 10*^−^*^6^	0.680	3.53	1.34 × 10^6^	0.13
		3d	8.2	3.16 × 10*^−^*^5^	0.950	20.4	19250	8.91 × 10*^−^*^6^	0.676	3.81	2.47 × 10^6^	0.25
		7d	8.9	3.29 × 10*^−^*^5^	0.947	20.9	28134	1.03 × 10*^−^*^5^	0.701	6.02	3.98 × 10^6^	0.39
		14d	11.3	3.24 × 10*^−^*^5^	0.950	21.4	54358	6.03 × 10*^−^*^6^	0.650	3.32	1.80 × 10^7^	0.34
6	Blank	1h	37.3	−	−	−	−	1.11 × 10*^−^*^3^	0.708	294	1049	3.9
		2d	35.5	−	−	−	−	1.84 × 10*^−^*^3^	0.750	730	938	8.8
		30d	38.0	−	−	−	−	4.00 × 10*^−^*^3^	0.600	1085	500	10
	0.5% LDH-NO_2_	1h	33.4	2.93 × 10*^−^*^5^	0.867	10.1	5063	3.46 × 10*^−^*^6^	0.925	2.50	1.21 × 10^6^	4.2
		20h	31.2	2.20 × 10*^−^*^5^	0.870	7.41	92184	2.27 × 10*^−^*^6^	0.898	1.89	5.30 × 10^6^	3.4
		2d	34.5	1.86 × 10*^−^*^5^	0.878	6.70	67366	3.44 × 10*^−^*^6^	0.814	2.45	9.21 × 10^6^	1.4
		30d	30.0	2.50 × 10*^−^*^5^	0.868	8.37	9089	3.40 × 10*^−^*^6^	0.915	2.46	1.30 × 10^6^	4
	0.5% LDH-NO_3_	1h	32.5	3.22 × 10*^−^*^4^	0.645	16.2	23.5	6.82 × 10*^−^*^4^	0.595	40.4	1347	3
		2d	35.0	1.84 × 10*^−^*^3^	0.681	381	41.1	2.43 × 10*^−^*^3^	0.733	940	925	1.4
		30d	35.5	1.92 × 10*^−^*^3^	0.680	382	32	2.89 × 10*^−^*^3^	0.707	1063	1002	8

Note: *Y*_0_ is the frequency-independent admittance of the CPE; *n* is the power of the CPE; subscripts pass and *dl* correspond to passive layer and double layer, respectively; χ is Chi-squared.

**Table 2:** Parameters determined by numerical fitting of the EIS spectra shown in Figure 8 with capacitances calculated using Equation (2).

System	Time	*R_pore_* (Ω cm^2^)	*Y_0,pass_* (F cm^−2^ s^n−1^)	*n_pass_*	*C_pass_* (μF cm^−2^)	*R_pass_* (Ω cm^2^)	*Y_0,dl_* (F cm^−2^ s^n−1^)	*n_dl_*	*C_dl_* (μF cm^−2^)	*R_ct_* (Ω cm^2^)	*Χ^2^*/10^−4^
Reference	1d	851	4.30 × 10*^−^*^5^	0.980	40.2	802721	−	−	−	−	2.1
	4d	518	1.19 × 10*^−^*^4^	0.810	61.6	30565	1.85 × 10*^−^*^4^	0.598	409	4.22 × 10^4^	5
	21d	589	1.24 × 10*^−^*^4^	0.845	75.2	6515	2.50 × 10*^−^*^4^	0.636	293	2.80 × 10^4^	0.9
	69d	661	1.59 × 10*^−^*^4^	0.774	78.6	3860	7.73 × 10*^−^*^4^	0.615	1457	4.61 × 10^4^	1
LDH-NO_3_	19d	468	1.07 × 10*^−^*^4^	0.854	63.9	75078	2.04 × 10*^−^*^4^	0.902	260	1.21 × 10^5^	16
	28d	524	1.22 × 10*^−^*^4^	0.790	58.2	39152	2.88 × 10*^−^*^5^	0.942	28.3	8.47 × 10^4^	1.8
	72d	481	1.97 × 10*^−^*^4^	0.730	81.4	15423	5.42 ×10*^−^*^5^	0.460	43.3	1.12 × 10^6^	2.4
LDH-NO_2_	4d	474	5.17 × 10*^−^*^5^	0.987	49.2	998436	−	−	−	−	2
	13d	444	1.12 × 10*^−^*^4^	0.876	73.3	402510	−	−	−	−	3
	20d	491	1.37 × 10*^−^*^4^	0.827	77.4	17441	4.56 × 10*^−^*^5^	0.658	36.5	8.00 × 10^4^	0.7
	64d	450	1.63 × 10*^−^*^4^	0.808	86.9	15241	3.40 × 10*^−^*^6^	0.936	40.2	1.79 × 10^5^	0.8

Note: *R_pore_* is the pore resistance in the equivalent circuit of Figure 9.
